# Oral Health-Related Quality of Life Among Non-institutionalized Elderly in Malaysia: A Teaching Hospital-Based Survey

**DOI:** 10.7759/cureus.56202

**Published:** 2024-03-14

**Authors:** Avenia Shammah Ramien, Amirul Arif Bin Azmi, Sethu Ravichandran, Trisha Thein Wai Li, Sashmeetavani Ravendran, Htoo Htoo Kyaw Soe, Ramanathan Ravi, Harini Priya, Silpa Madhuri Chikkala, Renjith George Pallivathukal

**Affiliations:** 1 Oral Pathology, Faculty of Dentistry, Manipal University College Malaysia, Melaka, MYS; 2 Community Medicine, Manipal University College Malaysia, Melaka, MYS; 3 Conservative Dentistry and Endodontics, Faculty of Dentistry, Manipal University College Malaysia, Melaka, MYS; 4 Oral and Maxillofacial Pathology, Manipal University College Malaysia, Melaka, MYS; 5 Prosthodontics, Manipal University College Malaysia, Melaka, MYS; 6 Oral Pathology and Microbiology, Manipal University College Malaysia, Melaka, MYS

**Keywords:** oral health survey, non-institutionalized, elderly population, oral-health-related quality of life, geriatric oral health assessment index

## Abstract

Background

In Malaysia, the Geriatric Oral Health Assessment Index (GOHAI) has been recognized as a vital instrument for evaluating oral health-related quality of life among the elderly population. Its integration into the National Health and Morbidity Survey (NHMS) in 2018 demonstrates the country's dedication to understanding and addressing the unique oral health challenges faced by older individuals. The NHMS, led by the Ministry of Health Malaysia, serves as a crucial platform for identifying and addressing healthcare needs, while also working towards achieving sustainable development goals. This study aimed to provide valuable information for stakeholders and researchers by investigating the relationship between quality of life related to oral health and demographic factors, with the ultimate goal of enhancing oral healthcare for older individuals.

Objective

The objective of this research was to identify the variables that impact the Oral Health-Related Quality of Life (OHRQoL) of non-institutionalized elderly individuals who visit the Klinik Pergigian Manipal University College Malaysia (MUCM). By utilizing the OHRQoL, dental practitioners can gain insight into the elderly's oral health-related quality of life, which is crucial information for dental healthcare providers to effectively reach out to and cater to the elderly at the institution.

Methodology

A cross-sectional design was employed, utilizing a non-probability sampling method to select eligible elderly individuals at the clinic. A validated questionnaire comprising 18 items covering sociodemographic details and the Geriatric Oral Health Assessment Index (GOHAI) was distributed to individuals above 60 years via printed forms. One way ANOVA, unpaired t-test and multiple linear regression analysis were performed to analyze the data.

Results

The overall mean GOHAI score among non-institutionalized elderly attending Klinik Pergigian MUCM was 48.38±9.33, indicating poor OHRQoL. The psychosocial impact domain had a mean score of 19.33±5.21, surpassing the pain and discomfort domain with a score of 10.73±2.82, highlighting the significant impact of psychosocial factors on poor oral health-related quality of life. Multiple linear regression analysis revealed no significant associations between OHRQoL and factors such as ethnicity, living arrangements apart from spouse and family, or tertiary education among older individuals after adjusting for confounding variables.

Conclusions

This study indicates that sociodemographic aspects have minimal impact on the OHRQoL of seniors. Further examination is needed to understand the economic aspects of tooth replacement options and preventive measures in this age group. To enhance the OHRQoL of older adults, especially those not living in facilities like nursing homes or assisted living centers, tailored oral healthcare plans and strategies are crucial. Interdisciplinary collaboration among mental health professionals, geriatric experts, and oral healthcare providers is crucial for empowering both healthcare practitioners and seniors to maintain optimal oral health.

## Introduction

Population aging is a global phenomenon driven by advancements in living conditions and medical care. Over time, health research has focused on extending life expectancy and promoting healthy aging. One crucial aspect of this is understanding the impact of oral health on individuals' quality of life [1-3].

Oral Health-Related Quality of Life (OHRQoL) is a multidimensional construct that encompasses subjective notions related to functional and emotional well-being, as well as satisfaction with health care. Studies have shown that in older adults, lower OHRQoL is associated with various factors such as socioeconomic disadvantage, negative self-rated oral health, reduced number of remaining teeth, irregular dental visits, poor chewing function, and inadequate nutritional condition [4-6]. A recent systematic review observed that the elderly group of the population had no proper oral health-related quality of life [3]. To tackle the increasing challenges of oral care in aging societies, it will be necessary to provide specialized oral health care for vulnerable older individuals and to integrate oral care with primary care services [7].

The pace of population aging is accelerating, particularly in developing countries like Malaysia. Malaysia, for instance, is on track to become an aged nation by 2030 due to longer life expectancy and a rapid decline in the total fertility rate. This demographic shift underscores the importance of understanding and addressing the oral health needs of older individuals [8-9].

In Malaysia, the Geriatric Oral Health Assessment Index (GOHAI) has been recognized as a valuable tool for assessing OHRQoL. Its adoption in the National Health and Morbidity Survey (NHMS) 2018 reflects a commitment to understanding the oral health challenges faced by older populations. The NHMS, conducted by the Ministry of Health Malaysia, serves as a platform for addressing healthcare needs and achieving sustainable development goals. By evaluating OHRQoL and its sociodemographic associations, this study aims to provide insights for stakeholders and researchers to improve oral health care for older individuals [10-11]. By focusing on older persons' health in the NHMS 2018, this study contributes to the broader effort of enhancing the well-being of aging populations. Consequently, findings from this research will inform policy decisions and guide future interventions aimed at improving oral health outcomes for older individuals.

## Materials and methods

Study design and sample size

The study employed a cross-sectional design, which involved data collection at a single point in time. The target population was determined based on the age distribution found in the patient records of Klinik Pergigian MUCM. Non-probability purposive sampling was used, with a focus on older patients who were non-institutionalized and attended the Klinik Pergigian MUCM. This method allowed for participants to be selected according to predetermined criteria. The required sample size for estimating a single population mean was determined based on a 95% confidence interval, a 0.5 margin of error, a 2.49 standard deviation [12], and an alpha of 0.05. The initial calculation indicated a minimum of 96 participants was necessary. However, accounting for a 10% dropout rate, the total number of participants needed was adjusted to 107.

The survey instrument used in this study was the Geriatric Oral Health Assessment Index (GOHAI; see appendix), which was created to assess both the psychological and functional effects of oral health on the quality of life of elderly individuals. This comprehensive instrument contains 12 items that are divided into three domains: physical function, comprising four questions, assessing aspects such as chewing, swallowing, and speaking comfortably; pain or discomfort, comprising three questions, assessing the presence and severity of oral pain or discomfort experienced by the individual; and psychosocial impacts, comprising five questions, focusing on the individual's perception of their oral health and its impact on their social interactions and self-image.

The assessments were conducted using a reference period of the past 12 months, providing a comprehensive overview of respondents' experiences. The GOHAI uses a six-point Likert scale for participant ratings. For data analysis purposes, questions three, five, and seven were rephrased in negative form, and the scoring was reversed. This modification aimed to ensure consistency and alignment between the scoring of negative and positive statements in the questionnaire, enhancing the accuracy and reliability of the instrument. The internal consistency of GOHAI was determined by calculating Cronbach's alpha coefficient, which resulted in a value of 0.689. The final score for each respondent ranged from zero to 60 points and was used to categorize individuals into three groups: good (score 57-60), fair (score 51-56), or poor (score ≤50). Additionally, the instrument included self-perception questions on general health, oral health, and oral healthcare utilization based on insights from previous studies. Overall, the GOHAI Malay version comprehensive survey instrument was designed to provide a detailed understanding of the oral health-related quality of life (QoL) among older individuals, considering various dimensions and incorporating self-perception assessments of general and oral health, as well as oral healthcare utilization.

Data collection

The study was approved by the Manipal University College Malaysia Human Research and Ethical Committee, and each participant was provided with a bilingual consent form outlining the study purpose in both Malay.

A printed questionnaire with consent forms was distributed to elderly patients undergoing treatment at Klinik Pergigian MUCM. GOHAI questionnaire was utilized as the standard procedure for obtaining pertinent data from participants. To ensure consistency and reliability in data collection, a series of slides was utilized to explain the study objectives and the administration of the GOHAI questionnaire to interviewers. The data collection process was initiated in June 2023 and concluded in January 2024 and was carried out face-to-face using the printed GOHAI questionnaire among elderly participants attending the Klinik Pergigian MUCM.

The statistical analysis was performed using SPSS software (IBM, Inc., Armonk, US) [13]. Descriptive statistics, such as frequency and percentage, were utilized to measure demographic variables, including age, gender, ethnicity, education level, living status, and income level. The mean was used to calculate the scores of the quantitative variable, which was the elderly's oral health-related quality of life, as measured by the GOHAI questionnaire. Statistical tests were utilized to calculate the association between the independent and dependent variables. Specifically, one-way ANOVA was used to evaluate differences between groups based on variables such as ethnicity, education level, living arrangement, and income level. An unpaired T-test was used to evaluate differences between groups based on variables such as sex, age, and self-perceived oral health status. Multiple linear regression analysis was performed to determine the relationship between independent variables of OHRQoL after adjusting the covariates. A 95% confidence level was calculated, and a p-value less than 0.05 was considered statistically significant.

## Results

The study recruited 99 of the 120 elderly individuals, achieving a response rate of 82.5%. Table 1 presents an overview of the sociodemographic characteristics of the older individuals who participated in the study. The majority of respondents were male (55.56%), aged between 60-70 years (47.47%), of Chinese ethnicity (68.69%), living with family/relatives (57.58%), with secondary education level (53.54%), and falling under the B40 income level category (74.75%). Table 2 shows the frequency of the domain/item scores. Elderly respondents commonly reported limitations in physical function related to biting or chewing (35.35%) and speaking clearly (58.59%), as well as experiencing discomfort such as sensitivity to hot, cold, or sweet foods (33.33%) and using medication to relieve pain (72.73%), indicating significant oral health-related quality of life issues.

**Table 1:** Demographic details of respondents RM - Ringgit Malaysia

Item	Variables	n (%)
Gender	Male	55 (55.56)
	Female	44 (44.44)
Age (years)	60-70	47 (47.47)
	70-80	45 (45.45)
	>81	7 (7.07)
Ethnicity	Malay	6 (6.06)
	Chinese	68 (68.69)
	Indian	23 (23.23)
	Other	2 (2.02)
Living status	With spouse/partner	31 (31.31)
	With family/relatives	57 (57.58)
	Others	11 (11.11)
Education level	Primary	21 (21.21)
	Secondary	53 (53.54)
	Tertiary	25 (25.25)
Income level	T20 (above RM 10970)	8 (8.08)
	M40 (RM 4851-10970)	17 (17.2)
	B40 (below RM 4850)	74 (74.75)

**Table 2:** Oral health-related quality of life of the elderly assessed by GOHAI GOHAI - Geriatric Oral Health Assessment Index

Domain	Item	Responses, n (%)					
		Always	Very often	Often	Sometimes	Seldom	Never
Physical function	Limit the kinds of food	13 (13.13)	13 (13.13)	12 (12.12)	18 (18.18)	14 (14.14)	29 (29.29)
	Trouble biting or chewing	15 (15.15)	12 (12.12)	8 (8.08)	16 (16.16)	13 (13.13)	35 (35.35)
	Able to swallow comfortably	54 (54.55)	10 (10.10)	4 (4.04)	2 (2.02)	7 (7.07)	22 (22.22)
	Unable to speak clearly	9 (9.09)	2 (2.02)	5 (5.05)	11 (11.11)	14 (14.14)	58 (58.59)
Pain and discomfort	Able to eat without discomfort	26 (26.26)	8 (8.08)	9 (9.09)	20 (20.20)	8 (8.08)	28 (28.28)
	Used medication to relieve pain	3 (3.03)	0 (0.0)	3 (3.03)	10 (10.10)	11 (11.11)	72 (72.73)
	Sensitive to hot, cold, or sweet foods	33 (33.33)	21 (21.21)	14 (14.14)	10 (10.10)	11 (11.11)	10 (10.10)
Psychosocial impacts	Limit contact with people	1 (1.01)	3 (3.03)	3 (3.03)	11 (11.11)	12 (12.12)	69 (69.70)
	Pleased with a look of the teeth	13 (13.13)	5 (5.05)	13 (13.13)	14 (14.14)	20 (20.20)	34 (34.34)
	Worried about teeth, gums, or dentures	4 (4.04)	3 (3.03)	6 (6.06)	9 (9.09)	20 (20.20)	57 (57.58)
	Self-conscious about teeth, gums, or dentures	3 (3.03)	6 (6.06)	4 (4.04)	8 (8.08)	6 (6.06)	72 (72.73)
	Uncomfortable eating in front of others	5 (5.05)	5 (5.05)	4 (4.04)	18 (18.18)	12 (12.12)	55 (55.56)

The results reveal that the majority of elderly individuals faced issues with most of the items related to the psychosocial impact domain, including limiting contact with people, being self-conscious or worried about their teeth, gums, or dentures, and feeling uncomfortable eating in front of others. The mean scores of the OHRQoL domains, assessed using the GOHAI, are presented in Table 3. The psychosocial impact domain demonstrated the highest mean score of 19.33±5.21 among the three domains of OHRQoL, which included physical function and pain and discomfort. The mean scores for physical function and pain and discomfort were 13.32±4.71 and 10.73±2.82, respectively. The overall mean±SD of the GOHAI score was 48.38±9.33. There were no statistically significant differences in the mean GOHAI scores among different sociodemographic variables, indicating consistent oral health-related quality of life across gender, age, ethnicity, living status, education level, and income level.

**Table 3:** Mean GOHAI score of OHRQoL domains GOHAI - Geriatric Oral Health Assessment Index; OHRQoL - Oral Health-Related Quality of Life

Domains	Mean score ± SD
Physical function (items 1 + 2 + 3 + 4)	13.32±4.71
Pain and discomfort (items 5 + 8 + 12)	10.73±2.82
Psychosocial impacts (items 6 + 7 + 8 + 10 +11)	19.33±5.21
Total mean GOHAI score	48.38±9.33

Table 4 illustrates that individuals aged 60-70 had the lowest mean GOHAI score of 42.83±8.88 compared to those aged 70-80 and above 81, who had mean GOHAI scores of 43.96±10.05 and 43.43±8.46, respectively. Gender, age, ethnicity, living status, education level, and income level were not significant predictors of OHRQoL, suggesting that these factors may not have a strong influence on individuals' oral health perceptions.

**Table 4:** Mean GOHAI score of respondents in relation to sociodemographic variables GOHAI - Geriatric Oral Health Assessment Index; RM - Ringgit Malaysia

Variables	n (%)	Mean GOHAI score ± SD	p-value
Gender			
Male	55 (55.56)	43.56±9.69	0.830
Female	44 (44.44)	43.16±8.97	
Age (years)			
60-70	47 (47.47)	42.83±8.88	0.849
70-80	52 (52.53)	43.96±10.05	
>81	7 (7.07)	43.43±8.46	
Ethnicity			
Malay	6 (6.06)	38.33±9.56	0.403
Chinese	68 (68.69)	43.60±9.21	
Indian	23 (23.23)	43.43±9.80	
Other	2 (2.02)	50.5±3.54	
Living status			
With spouse/partner	31 (31.31)	43.00±9.87	0.821
With family/relatives	57 (57.58)	43.84±8.78	
Others	11 (11.11)	42.09±11.20	
Education level			
Primary	21 (21.21)	44.52±9.37	0.699
Secondary	53 (53.54)	42.66±9.64	
Tertiary	25 (25.25)	43.96±8.87	
Income level			
T20 (above RM 10970)	8 (8.08)	40.88±9.01	0.588
M40 (RM 4851-10970)	17 (17.2)	42.24±10.41	
B40 (below RM 4850)	74 (74.75)	43.92±9.17	

The lowest mean GOHAI score was recorded among participants of Malay ethnicity (38.33±9.56), while the "others" category had the highest mean GOHAI score (50.5±3.54). Elderly individuals who lived with family members/relatives had a higher mean GOHAI score (43.84±8.78) than those who lived with a spouse/partner (43.00±9.87). Participants with lower education levels (44.52±9.37) and those with a monthly income <Ringgit Malaysia (RM) 4850 (43.92±9.17) recorded the highest mean GOHAI score. However, there were no significant associations between sociodemographic variables and OHRQoL.

Table 5 displays the results of multiple linear regression analysis that considers the factors associated with OHRQoL after adjusting for confounding variables. Unfortunately, due to the limited sample size, no significant results were obtained from this analysis. Gender, age, ethnicity, living status, education level, and income level were not significant predictors of OHRQoL, suggesting that these factors may not have a strong influence on individuals' oral health perceptions. However, each variable still produced reliable data for discussion purposes. The mean GOHAI score for elderly male participants was 0.973, which was higher than that of female participants.

**Table 5:** Multiple linear regression of factors associated with OHRQoL OHRQoL - Oral Health-Related Quality of Life; RM - Ringgit Malaysia

Variables		Unstandardized beta coefficient	95% CI	Standardized beta coefficient	p-value
Gender	Female	Reference			
	Male	0.973	-3.347, 5.293	0.052	0.655
Age (years)	>81	Reference			
	60-70	0.376	-8.136, 8.888	0.02	0.93
	70-80	-0.109	-8.398, 8.179	-0.006	0.979
Ethnicity	Malay	Reference			
	Chinese	5.98	-2.560, 14.521	0.299	0.168
	Indian	5.986	-3.289, 15.260	0.272	0.203
	Other	12.859	-3.839, 29.557	0.195	0.129
Living status	With family/relatives	Reference			
	With spouse/partner	-1.376	- 6.098, 3.345	-0.069	0.564
	Others	-4.857	-11.802, 2.087	-0.164	0.168
Education level	Primary	Reference			
	Secondary	-2.8	-8.017, 2.416	-0.15	0.289
	Tertiary	0.676	-6.021, 7.373	0.032	0.841
Income level	B40 (below RM 4850)	Reference			
	M40 (RM 4851-10970)	-3.666	-9.605, 2.274	-0.149	0.223
	T20 (above RM 10970)	-4.737	-12.585, 3.110	-0.139	0.233

The participants aged >81 years were selected as the reference group. Elderly participants aged 60-70 had a mean GOHAI score of 0.376 more than the reference age group, whereas elderly aged 70-80 had a mean GOHAI score of 0.109 less than the reference age group. Elderly Malay individuals were selected as the reference group. In comparison, older individuals of the Chinese, Indian, and other ethnicities showed a higher mean GOHAI score than the reference ethnicity group, wherein the elderly participants of the other ethnicity showed the highest mean GOHAI score difference of 12.859. Older persons living with family or relatives were selected as the reference group for the current living status variable. Elderly persons living with their spouse/partner showed a mean GOHAI score of 1.376, less than that of the reference group, while elderly participants living with others showed a mean GOHAI score of 4.857, which was less than that of the reference group.

Elderly participants with a primary education were designated as the reference group for the education variable. Compared to the reference group, elderly participants with secondary education had a mean GOHAI score that was 2.8 points lower, while those with tertiary education had a mean GOHAI score that was 0.676 points higher. Similarly, elderly participants with a household monthly income below RM 4850 were selected as the reference group for the household monthly income variable. Elderly participants from the M40 (RM 4851-10970) and T20 (above RM 10970) income level groups had a mean GOHAI score that was lower than the reference group, while those in the T20 group had the lowest mean GOHAI score difference of 4.73. It is worth noting that the p-value for the factors associated with OHRQoL was greater than 0.05, indicating that they were not statistically significant.

## Discussion

According to the results, elderly individuals who attended Klinik Pergigian Manipal University College (who are not residing in any institutionalized settings like a nursing home or assisted living centers) demonstrated a poor perception of their oral health status, as evidenced by their low GOHAI scores, which had a mean of 48.38. This indicates that their oral health had a negative impact on their quality of life, a finding that is consistent with study results done by Tenani et al. (2021) [14]. In 2020, a national survey conducted by Mohamad Fuad et al. [12] revealed that the overall GOHAI score in Malaysia was considered fair and was higher than that of other Asian countries. Furthermore, studies on elderly individuals using the GOHAI internationally have shown that the mean GOHAI score of the elderly in this study was higher than Mexico (46.8±6.2), as reported by Montes-Cruz et al. (2014) [15]; however, it was lower than that reported by China (48.9±7.2) in 2002 [16], Brazil (53.9), as reported by de Andrade et al. (2012) [17], and Germany (51.9±7.6), as reported by Pistorius et al. (2013) [18]. According to Nikbin et al. (2014) [19], individuals from different cultural backgrounds may have varying responses to statements in the GOHAI questionnaire. Given the increasing interest in research exploring self-perception of health as a means of understanding and monitoring it, which has also been increasingly recommended by the World Health Organization (WHO), OHRQoL is a crucial aspect that needs to be continuously monitored in every discussion regarding an individual's quality of life. Therefore, this study was conducted to provide fresh insights into this domain and to provide valuable data and understanding of the needs of the population for the benefit of the state of Melaka.

Our study revealed that there was no significant association between the ethnicity of elderly individuals and their OHRQoL, as measured by the GOHAI score. However, when comparing the mean GOHAI score of the Chinese and Indian ethnicities to the Malay ethnicity, it was found that the Chinese and Indian ethnicities had a higher mean GOHAI score than the Malay ethnicity. This finding is consistent with a study conducted by Othman et al. (2021) [20] which showed that the Malay ethnicity had one of the lowest GOHAI scores when compared to the Chinese but differed from Indians, whereby Indians had an even slightly lower GOHAI score. However, it is important to note that there is currently no published evidence supporting an association between ethnicity and GOHAI score, as stated by Mohammad Fuad et al. (2020) [12]. Therefore, further research is needed to explore the relationship between ethnicity and OHRQoL.

In terms of the living status of elderly individuals, our study found no significant association between their living status and their mean GOHAI scores. However, our results showed that elderly individuals living with their spouse/partner or others had lower GOHAI scores than those living with family. This could be due to older adults relying on their family members for assistance in accessing healthcare services, as found in a study conducted in India by Dable et al. (2013) [21]. As their partners may also be elderly, they may not be able to rely on them for regular treatment, which could lead to similar challenges in the Malaysian population.

The outcomes of this study reveal that the monthly income of older individuals has no statistically significant association with their GOHAI scores. However, when contrasted with the constant of B40, the results indicate that elderly individuals belonging to the M40 and T20 categories of income tend to have better OHRQoL. This finding contradicts the results of previous studies, which generally indicate a significant connection between income level and GOHAI scores [20]. According to a review by Janto et al. (2022) [22], barriers such as the inability to perceive the need to visit the dentist, fear, anxiety, past negative experiences, and lack of awareness of dental problems can contribute to the low mean GOHAI scores of elderly individuals.

As stated by Mohamad Fuad et al. (2020) [12], gender has been commonly believed to influence oral health and OHRQoL; however, this study did not find any significant difference between elderly males and females. These findings are consistent with those of a smaller study conducted in local villages byOthman et al. (2021) [20]​​​​​. Our results also support this, as gender had little to no impact on oral health perception. This finding is contradicted by a study conducted by Sfeatcu et al. (2022) [23], which showed that female subjects have positive attitudes towards medical professional care, invest more in oral care, and have more oral health knowledge than men. 

Our study showed that age had no significant association with oral health. However, several studies have shown or proved otherwise that lower mental status and lower self-rated health were associated with being at risk for oral health [24]. This may be due to the deteriorating mental condition associated with older age. Another study by Ahmad et al. (2018) suggested an inverse relationship between regular oral healthcare utilization and increasing age. In their study, working-age adults (18-59 years) were more likely to utilize oral healthcare than older adults (≥60 years). Oral healthcare utilization among the elderly was also still low despite various efforts, with 14.7% of Malaysians aged 60 years and above who had never been to a dentist [25,26].

This study showed that the educational level of the elderly was not significantly associated with OHRQoL. The findings of this study contradict those of previous studies, as most of them showed a significant association between education level and GOHAI scores, wherein individuals with lower education levels were more likely to have poor OHRQoL. This could be due to the fact that oral health education has not been reinforced for these individuals from their early days; hence, maintenance of their oral health has not been made a priority in their daily lives and may worsen as a result of aging. According to Kementerian Kesihatan Malaysia, a 2022 report from the Oral Health Programme showed that the operational budget allocation was approximately 2-3% of the entire Ministry of Health (MOH) budget [27]. This has resulted in major financial strain on the public healthcare system, as 79.5% of Malaysians seek oral healthcare treatments at public healthcare facilities [28]. Thus, the respective authorities may have found it difficult to organize widespread outreach programs across Malaysia to educate the elderly about their oral health.

Limitations

The findings of this study offer valuable insights, but it is important to acknowledge the limitations, such as the cross-sectional design and reliance on self-reported data. The sample was selected using a non-probability sampling method, which may have contributed to the imbalance in the amount of data collected from different ethnicities and income levels. The small sample size, limited to those attending Klinik Pergigian Manipal University College instead of a local government clinic, prevented the use of other sampling methods that could have addressed this limitation. Future research should explore longitudinal associations and incorporate clinical assessments to enhance the robustness of these findings. Additionally, qualitative investigations may uncover nuanced factors that contribute to psychosocial impacts on OHRQoL.

## Conclusions

This research concludes that sociodemographic factors do not have a substantial impact on the oral health-related quality of life (OHRQoL) of elderly individuals. Further investigation is needed to better understand the economic aspects of tooth replacement options and preventive interventions for this population. To improve the OHRQoL, customized oral healthcare plans and strategies are necessary, particularly for non-institutionalized older adults. This study provides important insights and recommends interdisciplinary collaboration between mental health professionals, geriatric specialists, and oral healthcare providers to empower healthcare practitioners and the elderly in managing optimal oral health. Given the poor OHRQoL among non-institutionalized elderly attendees at the Klinik Pergigian MUCM, targeted public health interventions are necessary. Community-based oral health programs, awareness campaigns, and culturally sensitive educational initiatives can help mitigate these disparities and improve the overall well-being of the vulnerable population. Overall, this study reiterates the significance of personalized oral healthcare programs in enhancing the well-being of the aging population in Malaysia.

## Appendices

**Table 6:** Geriatric Oral Health Assessment Index (GOHAI) The survey tool utilized in this study was the Geriatric Oral Health Assessment Index (GOHAI), designed explicitly to assess the psychological and functional effects of oral health on the quality of life of older adults.

A. PATIENT BIODATA / BIODATA PESAKIT		
#	Variable and question (in Eglish / Malay)	Responses (in Eglish / Malay)
1	Gender / Jantina	Female / Perempuan
		Male / Lelaki
2	Age / Umur	60-70 years old / 60-70 tahun
		70-80 years old / 70-80 tahun
		>81 years old / >81 tahun
3	Ethnicity / Keturunan	Indian
		Chinese
		Malay
		Others
4	What is your current living status? / Pada masa ini, anda tinggal bersama?	Spouse/partner / isteri/suami
		family/relatives / keluarga/saudara mara
		Others / Lain - Lain
5	What is your educational level? / Apakah tahap pendidikan anda?	No education / Tiada pendidikan
		Primary / Pendidikan rendah
		Secondary / Pendidikan menengah
		Tertiary / Pengajian tinggi
6	What is your monthly household income? / Berapakah pendapatan isi rumah bulanan anda?	below RM 4,850
		RM 4,851 to RM 10,970
		more than RM 10,971
B. ORAL HEALTH-RELATED QUALITY OF LIFE / KUALITI HIDUP BERKAITAN KESIHATAN MULUT		
7	How often did you limit the kinds or amounts of food you eat because of problems with your teeth or dentures? / Berapa kerapkah anda menghadkan jenis atau jumlah makanan yang anda makan disebabkan masalah gigi atau gigi palsu anda?	Always/ Sentiasa; Very often/ Sangat kerap; Often/ Kerap; Sometimes/ Kadang-kadang; Seldom/ Jarang sekali; Never/ Tidak pernah
8	How often did you have trouble biting or chewing any kinds of food, such as firm meat or apples? / Berapa kerapkah anda mengalami kesukaran menggigit atau mengunyah sebarang jenis makanan pejal misalnya daging yang liat,atau buah epal?	
9	How often were you able to swallow comfortably? / Berapa kerapkah anda boleh menelan dengan mudah?	
10	How often have your teeth or dentures prevented you from speaking the way you wanted? / Berapa kerapkah gigi atau gigi palsu anda menghalang anda daripada bercakap dengan cara yang diingini?	
11	Have you felt discomfort due to food getting stuck in between your teeth, mouth or dentures? / Berapa kerapkah anda boleh memakan apa sahaja tanpa kesukaran?	
12	How often did you limit contacts with people because of the condition of your teeth or dentures? / Pernahkan anda mengelak diri dari bertemu dengan orang lain disebabkan keadaan gigi atau gigi palsu anda?	
13	How often were you pleased or happy with the looks of your teeth and gums, or dentures? / Berapa kerapkah anda berpuas hati dengan rupa gigi dan gusi, atau gigi palsu anda?	
14	How often did you use medication to relieve pain or discomfort from around your mouth? / Pernahkah anda mengguna atau memakan ubat untuk melegakan sakit atau rasa tidak selesa di kawasan mulut anda?	
15	How often were you worried or concerned about the problems with your teeth, gums or dentures? / Pernahkah anda berasa risau atau bimbang tentang masalah gigi, gusi atau gigi palsu anda?	
16	How often did you feel nervous or self-conscious because of problems with your teeth, gums or dentures? / Pernahkah anda berasa malu atau gelisah kerana masalah gigi, gusi atau gigi palsu anda?	
17	How often did you feel uncomfortable eating in front of people because of problems with your teeth or dentures? / Pernahkah anda berasa tidak selesa apabila makan bersama orang lain disebabkan masalah gigi atau gigi palsu anda?	
18	How often were your teeth or gums sensitive to hot, cold or sweet? / Pernahkah gigi atau gusi anda terasa ngilu atau sengal apabila makan/minum benda yang panas, sejuk atau manis?	
